# Double-Quantum-Well AlGaN/GaN Field Effect Transistors with Top and Back Gates: Electrical and Noise Characteristics

**DOI:** 10.3390/mi12060721

**Published:** 2021-06-19

**Authors:** Maksym Dub, Pavlo Sai, Maciej Sakowicz, Lukasz Janicki, Dmytro B. But, Paweł Prystawko, Grzegorz Cywiński, Wojciech Knap, Sergey Rumyantsev

**Affiliations:** 1 Center for Terahertz Research and Applications (CENTERA) Laboratories, Institute of High Pressure Physics PAS, ul. Sokołowska 29/37, 01-142 Warsaw, Poland; sajpasha@gmail.com (P.S.); but.dmitry@gmail.com (D.B.B.); gc@unipress.waw.pl (G.C.); knap.wojciech@gmail.com (W.K.); roumis4@gmail.com (S.R.); 2 Institute of High Pressure Physics PAS, ul. Sokołowska 29/37, 01-142 Warsaw, Poland; sakowicz@unipress.waw.pl (M.S.); pprysta@unipress.waw.pl (P.P.); 3 V. E. Lashkaryov Institute of Semiconductor Physics, National Academy of Sciences of Ukraine, 41 pr. Nauki, 03680 Kyiv, Ukraine; 4 Department of Semiconductor Materials Engineering, Wrocław University of Science and Technology, Wybrzeże Wyspiańskiego 27, 50-370 Wrocław, Poland; lukasz.janicki@pwr.edu.pl; 5 Centrum Zaawansowanych Materiałów i Technologii (CEZAMAT), Warsaw University of Technology, 02-822 Warsaw, Poland; 6 Laboratoire Charles Coulomb, University of Montpellier and CNRS UMR 5221, 34950 Montpellier, France

**Keywords:** AlGaN/GaN, quantum wells, grating gate, high electron mobility transistors

## Abstract

AlGaN/GaN fin-shaped and large-area grating gate transistors with two layers of two-dimensional electron gas and a back gate were fabricated and studied experimentally. The back gate allowed reducing the subthreshold leakage current, improving the subthreshold slope and adjusting the threshold voltage. At a certain back gate voltage, transistors operated as normally-off devices. Grating gate transistors with a high gate area demonstrated little subthreshold leakage current, which could be further reduced by the back gate. The low frequency noise measurements indicated identical noise properties and the same trap density responsible for noise when the transistors were controlled by either top or back gates. This result was explained by the tunneling of electrons to the traps in AlGaN as the main noise mechanism. The trap density extracted from the noise measurements was similar or less than that reported in the majority of publications on regular AlGaN/GaN transistors.

## 1. Introduction

Aluminum gallium nitride/gallium nitride (AlGaN/GaN) high electron mobility transistors (HEMTs) are already commercialized and are widely used in high-power switching and high-frequency applications. Unique and specific features of the AlGaN/GaN system are its high breakdown electric field and high electron concentration at the AlGaN/GaN interface, which is about an order of magnitude higher than that in an AlGaAs/GaAs system. Along with its reasonably high electron mobility, these properties provide superior characteristics to AlGaN/GaN HEMTs [1,2].

Since the first demonstration of an AlGaN/GaN HEMT at the beginning of the 1990s [3,4], there have been many attempts to further improve the transistor’s characteristics. Among them are devices consisting of double or multi-quantum well structures and devices with both top and back gates. Indeed, it was demonstrated that the carrying capability could be enhanced using multichannel structures [5,6,7,8,9,10,11]. Structures with the back gate were shown to exhibit enhanced transconductance and higher breakdown voltage. The short channel and current collapse effects can be also suppressed in these transistor’s structures [12,13,14,15,16].

Another approach, which is rarely used, is exploring the double-quantum-well structures when the bottom quantum well serves as a back gate to the top quantum well. We are aware only of a few publications from one group which attempted this approach [17,18].

Importantly, double-channel, high electron mobility structures with top grating gates have demonstrated a number of new effects at terahertz frequencies, which are promising for detectors, intensity modulators and filters [19,20]. These devices have to have large active areas, orders of magnitude larger than those of "standard" field effect transistors. This poses a serious challenge for fabricating such devices with low gate leakage current.

Another serious issue is the noise, which limits the performance of almost every electronic device. Although low and high frequency noise in AlGaN/GaN HEMTs were studied in many publications (see, for example, [21,22,23,24,25] and references therein), we are not aware of noise studies in double-quantum-well AlGaN/GaN HEMTs. The effect of the back gate on noise in single-quantum-well HEMTs was studied in [26]. It was shown that noise is strongly modified by the gates and the dependencies of noise on top and back gates are different. The effect was explained by different electron density distributions when voltage was applied to top and back gates. This makes noise studies in the double-quantum-well structures of special interest.

In this work we studied the feasibility of fabricating the double-quantum-well AlGaN/GaN structures of HEMTs with a top Schottky barrier gate and a back gate. Regular fin-shaped and large-area grating gate transistors were fabricated and studied. The studied DC characteristics of the structures showed that this kind of device works as intended, and the noise level is low. The major challenge in the fabrication of the grating gate devices is the gate leakage current which might be high in structures with large gate areas. It was shown that the back gate reduces the gate leakage current.

## 2. Device Fabrication and Experimental Details

The heterostructures were grown by metalorganic vapor phase epitaxy on ammono-GaN substrates. The structures consisted of 0.5 nm GaN cap, a 7 nm Al${}_{0.15}$Ga${}_{0.85}$N barrier layer, a 1.3 nm Al${}_{0.66}$Ga${}_{0.34}$N, a 348 nm intentionally undoped GaN, a 3 nm GaN cap, a 4.7 nm Al${}_{0.66}$Ga${}_{0.34}$N, a 200 nm GaN:Si (n = 6 × $10^{17}$ cm${}^{-3}$), a 400 nm GaN:Si (n = 6 × $10^{19}$ cm${}^{-3}$), and a 400 µm Ammono-GaN (n = 1.5 × $10^{19}$ cm${}^{-3}$). The top layers in this structure were the typical ones used in fabrication of AlGaN/GaN high electron motility transistors. The buried 4.7 nm Al${}_{0.66}$Ga${}_{0.34}$N and 200 nm GaN:Si layers provided the second conducting layer at a depth of ∼360 nm.

The device processing was performed using a commercial laser writer system for the lithography process with the 405 nm GaN laser source. At the first step of sample processing, the shallow 100 nm mesas were etched by inductively coupled plasma reactive ion etching. These mesas formed the channel of the transistors and isolated devices from each other. Ohmic contacts were fabricated using the regrowth technique [27], which included etching of 50 nm deep tranches at drain and source locations and growing there the heavily doped GaN islands. Then, Ti/Al/Ni/Au (150/1000/400/500 Å) metal stacks were deposited and annealed at 400 ${}^{{}^{\circ}}$C in a nitrogen atmosphere for 60 s. This technique allowed us the relatively low temperature of annealing which preserved the properties of the heterostructures. Measurements of the contact resistance using the transmission line method yielded the contacts resistances within the range of 0.1–0.5 Ω·mm. Despite the relatively high spread of the contact resistance, the total contact resistance of the studied transistors was always significantly smaller than the total drain-to-source resistance.

Then, the 700 nm deep trenches were etched in order to access the bottom conducting layer for the back gate contacts’ fabrication. Identically to drain and source contacts, metal stacks of Ti/Al/Ni/Au were deposited in the trenches and rapidly thermal annealed at 400 ${}^{{}^{\circ}}$C, in a nitrogen atmosphere for 60 s. The Schottky contacts for the top gate were formed by Ni/Au (150/350 Å).

**Figure 1:**
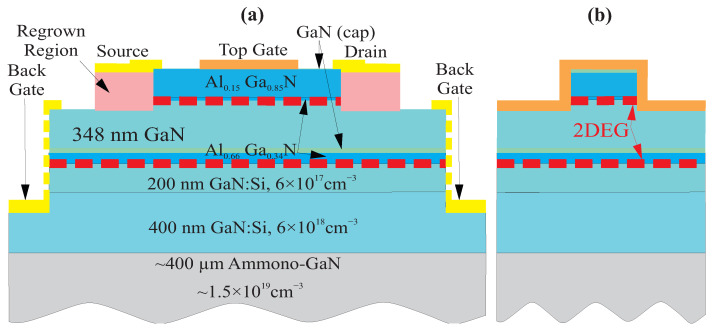
Cross -section views (not in scale) from source to drain (**a**) and perpendicular to it for fabricated AlGaN/GaN fin-shaped high electron mobility transistors (FinHEMTs) (**b**).

**Figure 2:**
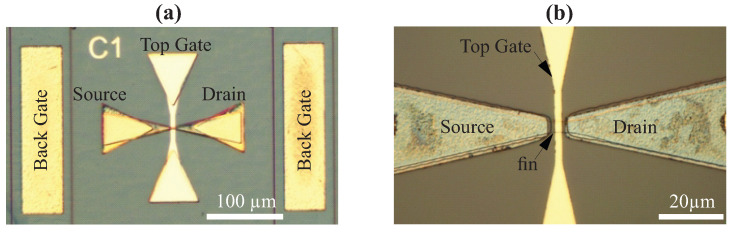
Optical microscope images of the fabricated FinHEMTs with back gate (**a**) and image of the active regions (**b**), channel width W${}_{Ch}$ = 5 µm and gate length L${}_{TG}$ = 2 µm.

Two types of transistors were fabricated. The first type of transistors was fin-shaped high electron mobility transistors (FinHEMTs). The schematic cross-sections in the direction from source (S) to drain (D) of the fabricated devices are shown in Figure 1a. Contacts of the back gate were made to the 400 nm GaN, which is shown by the yellow rectangles in Figure 1a. A cross-section in the direction perpendicular to the S–D direction is shown in Figure 1b. The gate length ranged from 2 to 10 µm with the gate width 5 µm. The optical microscopy images with different magnifications for one of the FinHEMTs are shown in Figure 2a,b.

**Figure 3:**
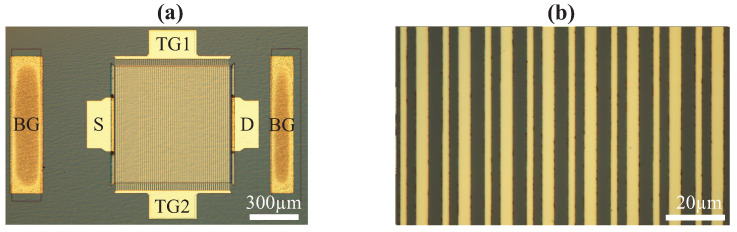
Optical microscope images of the fabricated large-area grating gate transistors (**a**) and close view of the gate structure (**b**).

The second type of transistor was the large-area grating gate transistor. Optical microscope images of one of the transistors at different levels of magnification are shown in Figure 3. The total transistor and gate areas were ∼0.64 and ∼0.25 mm${}^{2}$, respectively; individual gate lengths were from 1 to 3 µm; and the total number of gates was 130. These dimensions were chosen for the optimal coupling in the experiments with terahertz radiation, which will be published elsewhere. The actual gate area and overall size of the transistors were many orders of magnitude higher than that for the "standard" transistors. In these transistors, contacts of the back gate covered also the vertical walls of the mesas, as shown in Figure 1a by the yellow dashed lines. The structure included two independent gate structures with different gate lengths, as seen in Figure 3b. These structures are of special interest for the study of the effects at terahertz frequencies in GaN-based and other material systems (see [28,29,30,31,32,33] and references therein).

The current–voltage characteristics and noise were measured at room temperature on a wafer using a probe station. For the noise measurements, the voltage fluctuations (S${}_{v}$) from the drain load resistance R${}_{L}$ were amplified by a low noise amplifier and fast Fourier transformed with a dynamic signal analyzer. The short-circuit current fluctuations were calculated as S${}_{I}$ = S${}_{v}$[(R${}_{L}$+R${}_{D}$)/(R${}_{L}$R${}_{D}$)]${}^{2}$, where R${}_{D}$ is the drain-to-source resistance. The band diagram and carrier distributions at different biases were simulated using nextnano${}^{++}$, a self-consistent 1D Schrödinger–Poisson model solver [34].

## 3. Results and Discussion

**Figure 4:**
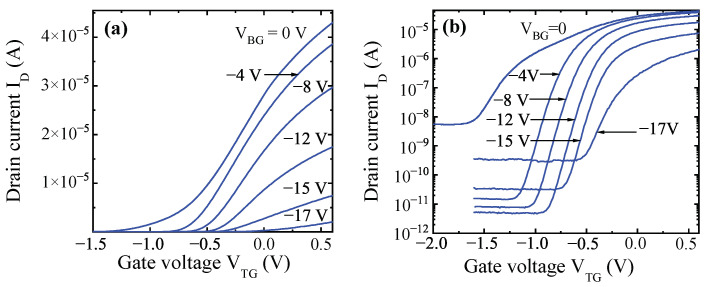
Transfer current–voltage characteristics of a FinHEMT at the linear (**a**) and semi-logarithmic scales (**b**) for one of the transistors with L${}_{TG}$ = 2 µm, W${}_{G}$ = 5 µm.

Figure 4 shows the top gate transfer current–voltage characteristics in the linear (a) and semi-logarithmic scales (b) for one of the studied devices measured at different values of back gate voltage. As seen, the back gate changed the threshold voltage, and at V${}_{BG}$ < –17 V the transistors operated as in a typical normally-off transistor. The subthreshold current was determined by the gate leakage current. It is shown in Figure 4 that back gate reduced the subthreshold current due to the reduction of the top gate leakage current. Only at high negative back gate voltage the leakage of the back gate starts to contribute to the subthreshold current.

**Figure 5:**
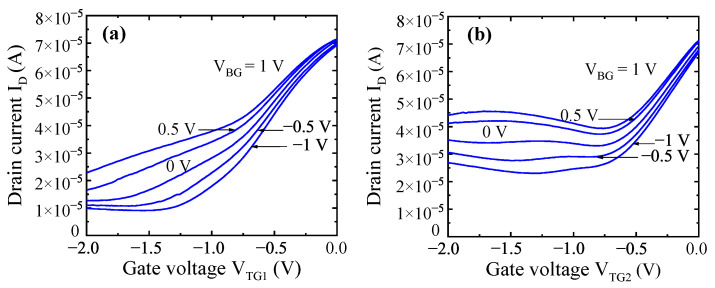
Drain current as a function of the gate voltage (transfer current–voltage characteristics) of a grating gate transistor when gate voltage V${}_{TG1}$ was applied to the first top gate with L${}_{G1}$ = 1.85 µm while keeping V${}_{TG2}$ = 0 V (**a**), and while varying V${}_{TG2}$ with L${}_{G2}$ = 3.7 µm and keeping V${}_{TG1}$ = 0 V (**b**). Measurements were taken at different back gate voltages V${}_{BG}$.

The grating gate transistors had two independent gate structures as shown in Figure 3b. L${}_{G1}$ = 1.85 µm, and L${}_{G2}$ = 3.7 µm. The total area of the channel was 0.64 mm${}^{2}$. The transistor had two independent gate structures, a total number of fingers of 114 and a total gate area of 0.25 mm${}^{2}$. Transfer current–voltage characteristics for one of the grating gate transistors for each gate are shown in Figure 5. Measurements were taken at different back gate voltages as functions of one of the top gate voltages while keeping the another one equal to zero.

One of the main challenges in fabricating this kind of large-gate-area transistor is achieving a small gate leakage current. As seen, the leakage current in subthreshold is in the range of tens of microamperes. Although this is a relatively high current, the gate voltage allows decreasing the drain current by about an order of magnitude. This was sufficient for the majority of experiments at terahertz frequencies, which will be reported elsewhere. It is important that the back gate voltage reduces the subthreshold leakage current.

**Figure 6:**
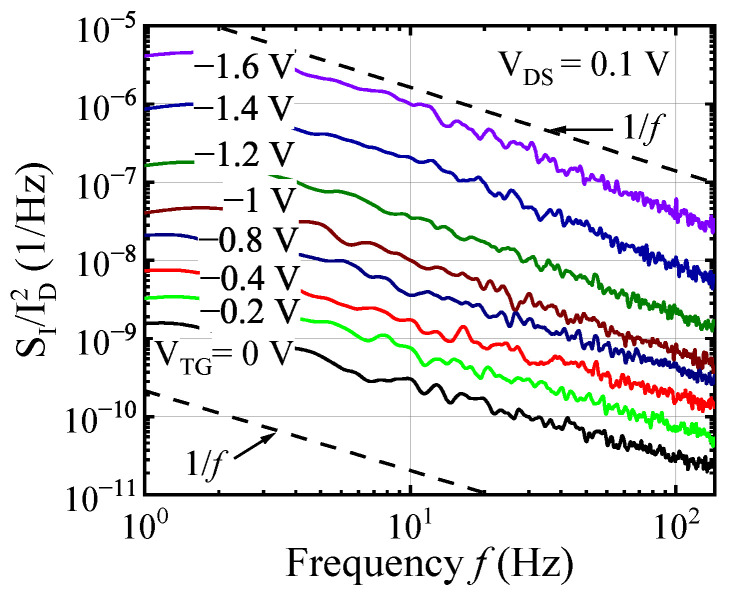
Noise spectra S${}_{I}/I_{D}^{2}$ for one of the FinHEMTs at drain V${}_{D}$ = 0.1 V, back gate voltage V${}_{BG}$ = 0 V and different top gate voltages V${}_{TG}$.

The low frequency noise was measured in FinHEMTs as a function of drain, top or back gate voltages in several devices of different dimensions. Figure 6 shows examples of the noise spectra of relative drain current fluctuations S${}_{I}$/I${}_{D}^{2}$ measured at fixed drain voltage and different top gate voltages. The noise spectra were always close to the 1/f${}^{\gamma }$ noise with the exponent γ=1.0−1.4. At constant gate voltages the spectral noise density of the drain current fluctuation, S${}_{I}$, was always proportional to the square of the drain current, S${}_{I}∼I_{D}^{2}$, indicating the absence of any current-induced effects. We did not notice systematic dependence of the noise spectra shape on the biases.

**Figure 7:**
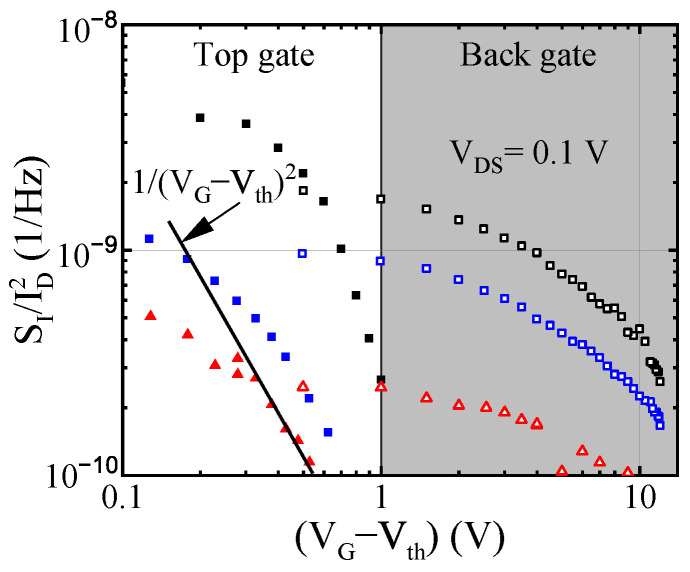
Spectral noise density S${}_{I}/I_{D}^{2}$ at frequency f = 10 Hz as a function of either top or back gate voltage swings (V${}_{G}-V_{th}$) for three transistors with different channel dimensions, while keeping the other gate voltage equal to zero. Filled symbols correspond to the top gate voltage; open symbols are for back gate voltage.

The dependencies of noise S${}_{I}/I_{D}^{2}$ as a function of the gate voltage swing (V${}_{G}$–V${}_{th}$) are shown in Figure 7 for one of the representative FinHEMTs. Here V${}_{G}$ is either top or back gate voltage and V${}_{th}$ is the threshold voltage, which is different for the top and back gates. The spectral noise density of the drain current fluctuations S${}_{I}/I_{D}^{2}$ in field effect transistors can be written in the following form:

(1) $$\frac{S_{I}}{I_{D}^{2}}=\frac{S_{R_{Ch}}}{R_{Ch}^{2}}\frac{R_{Ch}^{2}}{{\left(R_{Ch}+R_{acc}\right)}^{2}}+\frac{S_{R_{acc}}}{R_{acc}^{2}}\frac{R_{acc}^{2}}{{\left(R_{Ch}+R_{acc}\right)}^{2}},$$

where *S*${}_{R_{Ch}}/R_{Ch}^{2}$ and *S*${}_{R_{acc}}/R_{acc}^{2}$ are the spectral noise densities of the channel and access resistance fluctuations; *R*${}_{Ch}$ is the channel resistance under the top gate; *R*${}_{acc}$ is the access resistance, which is the sum of contact and channel resistances.

At high gate voltage, when the channel resistance is small, the second term in Equation (1) may dominate. As a result, it was observed in several publications [35,36] that noise slowly increases with the increase of gate voltage (decrease of *R*${}_{Ch}$). Since that was not the case in the studied devices, we conclude that the contact noise and noise of the ungated parts of the transistor did not contribute much to the overall drain current noise. The low frequency noise in field effect transistors, including GaN-based ones, often complies with the McWhorter model [37,38]. In the McWhorter model, the spectral noise density of channel resistance fluctuations in the linear regime is given by:

(2) $$\frac{S_{R_{Ch}}}{R_{Ch}^{2}}=\frac{k_{B}TN_{t}}{\alpha fW_{Ch}L_{G}n_{s}^{2}},$$

where k${}_{B}$ is the Boltzmann constant, *T* is the temperature, *N*${}_{t}$ is the effective trap density, *f* is the frequency, *W*${}_{Ch}$*L*${}_{G}$ is the gated channel area, *n*${}_{s}$ is the concentration and α is the attenuation coefficient of the electron wave function under the barrier. The estimate for the α value in the GaN/AlGaN system yields $\alpha ≅0.45\times 10^{8}$ cm${}^{-1}$ [38]. The channel concentration in the linear regime is *n*${}_{s}$ = C(V${}_{G}$–V${}_{th}$)/q, where C is the capacitance per unit gate area, V${}_{th}$ is the threshold voltage and q is the elemental charge. Therefore, as follows from Equations (1) and (2), the spectral noise density should depend on the gate voltage as S${}_{I}$/I${}_{D}^{2}$∼ (V${}_{G}$ – V${}_{th}$)${}^{-2}$, provided *R*${}_{Ch}\gg$ *R*${}_{acc}$. Steeper dependence of noise S${}_{I}/I_{D}^{2}$ on (V${}_{G}$–V${}_{th}$) can be a result of comparable values of *R*${}_{Ch}$ and *R*${}_{acc}$, and dependence of the trap density *N*${}_{t}$ on the Fermi level position, i.e., on the gate voltage.

**Figure 8:**
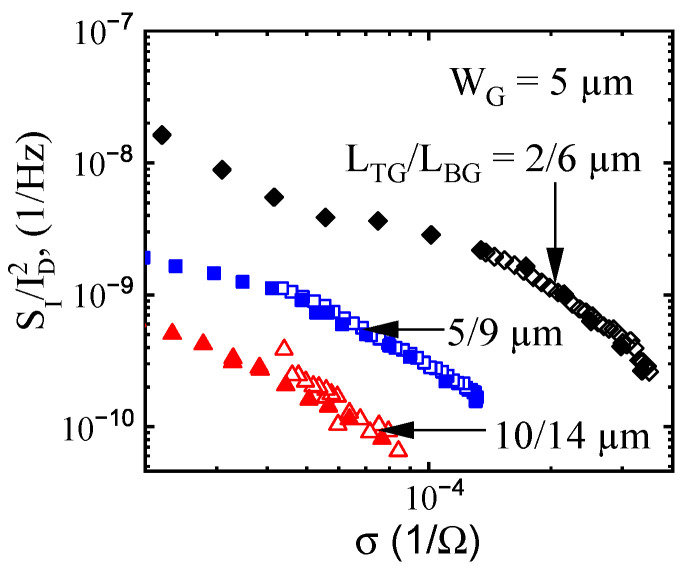
Spectral noise density S${}_{I}/I_{D}^{2}$ at frequency f = 10 Hz as a function of conductivity when controlled by the top or back gate voltages for three transistors with different channel dimensions. Filled symbols correspond to the top gate voltage; open symbols are for back gate voltage.

In order to compare the noise behavior when transistors were controlled by the top and back gate voltages, respectively, we plotted noise as a function of the channel conductivity σ. In Figure 8 the spectral noise density S${}_{I}$/I${}_{D}^{2}$ is shown as a function of conductivity for three transistors. Open symbols were obtained by varying back gate voltage, V${}_{BG}$, and maintaining top gate voltage V${}_{TG}$ = 0 V. Filled symbols correspond to the opposite situation when V${}_{BG}$ = 0 V and top gate voltage V${}_{TG}$ varies. When the channel conductivity is adjusted by either top or bottom gates, the band diagrams are completely different. However, both procedures resulted in virtually identical noise levels for the same values of channel conductivity.

Similar noise measurements as functions of the top or back gate voltages in [26] showed that noise behaves differently when drain current is adjusted by the top or back gate voltages. In particular, negative voltage applied to the back gate strongly decreased the noise. Different noise behavior when either top or back gate voltages were applied was explained by different electron density distribution in the channel. It was assumed that noise originates from the traps in GaN buffer, and that a negative back gate voltage pushes electrons from the buffer layer and reduces noise.

**Figure 9:**
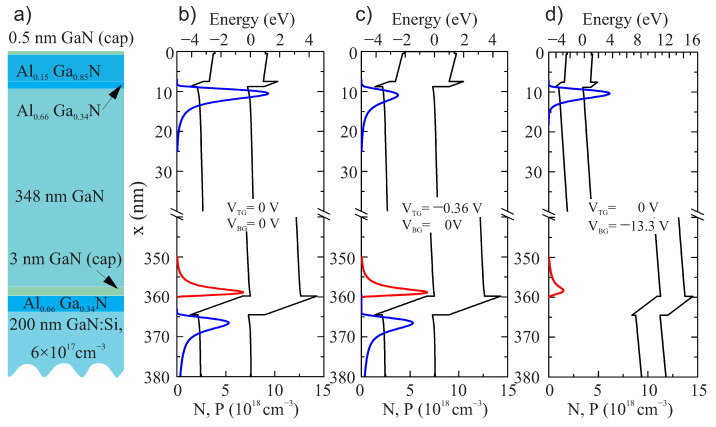
Layer sequence (**a**), band diagrams and carriers distributions at different configurations of the gate voltages (**b**–**d**). Gate voltages at (**c**,**d**) were adjusted to have approximately the same two-dimensional electron densities in the top conducting layer $n_{s}=1.8\times 10^{12}$ cm${}^{-3}$.

In order to verify this hypothesis, we modeled the band diagram and carrier distributions in the studied AlGaN/GaN structures at different gate voltages using the actual parameters of individual layers. The top channel was kept grounded throughout the calculations. Figure 9 shows the results of the simulations along with layers structure. Electron and hole density distributions N(*x*) and P(*x*) are shown with blue and red lines, respectively.

As seen from Figure 9b, at zero top and back gate voltages, there are two conducting layers. The bottom conducting layer consists of p-type and n-type closely spaced layers. Figure 9c,d shows the band diagrams and carrier distributions at V${}_{BG}$ = −13.3 V and V${}_{TG}$ = 0 V; and V${}_{BG}$ = 0 V and V${}_{TG}$ = −0.36 V, respectively. Both these gate voltage configurations correspond to approximately the same two-dimensional electron density, *n*${}_{s}$, equal to half of it at all zero biases. Indeed, the electron distributions in the top conducting layer are different for these two situations. The electron distribution at V${}_{BG}$ = −13.3 V, V${}_{TG}$ = 0 V, is more narrow. When only top gate voltage was applied, i.e., V${}_{BG}$ = 0 V, V${}_{TG}$ = −0.36 V, electrons spread out more in the GaN buffer. This is exactly the effect that was adopted in [26] in order to explain the noise reduction with a negative back gate voltage. Despite approximately the same two-dimensional concentration, the concentration per volume is different in Figure 9c,d. If noise originates from the trap inside the quantum well, it should depend on volume concentration as 1/*N*${}^{2}$ [39] and be different for these two situations. The bigger spread of electrons distribution function in the GaN layer in Figure 9c should result in higher noise if we assume that the noise originates from the tunneling to the traps in GaN layer away from the channel. Identical noise properties while operating top or bottom gates in the present study is an indication that in the structures fabricated, noise travels from the tunneling to the traps in AlGaN and not to the traps in GaN, as was assumed in [26].

The McWhorter model allows extracting the trap density responsible for the noise. Originally, the McWhorter model was developed for Si MOSFETs. In accordance with the model, the 1/f noise originates from the tunneling of the carriers to the gate dielectric. A similar mechanism of tunneling to the barrier AlGaN layer might be responsible for the 1/f noise in AlGaN/GaN HEMTs. However, there is no direct proof of this model’s validity for these devices. Therefore, the trap density, *N*${}_{t}$, extracted using the McWhorter model, should be considered as a figure of merit for the noise level and overall quality of the transistors. Since we consider the trap density *N*${}_{t}$ as a figure of merit, we did not take into account further complications of the model, which include, in particular, possible correlated mobility fluctuations. While calculating the trap density using Equation (2), one has to take into account the access resistance, i.e., Equation (1). The effect of the access resistance can be taken into account using the input gate voltage *S*${}_{V_{G}}$ for the trap density calculation [35]:

(3) $$S_{V_{G}}=\left(\frac{S_{I}}{I_{D}^{2}}\right)\left(\frac{I_{D}^{2}}{g_{m}^{2}}\right)=\frac{k_{B}TN_{t}q^{2}}{\alpha fW_{Ch}L_{G}C^{2}}.$$

We found *N*${}_{t}$ = (2–5) × 10${}^{19}$ eV${}^{-1}$cm${}^{-3}$. Although smaller values of the trap density were reported in a few publications [40,41], the majority of papers reported the trap density to be within the same range or higher [35,36,42,43,44].

## 4. Conclusions

AlGaN/GaN HEMTs with two parallel conducting layers and the back gate were fabricated and studied experimentally. It was demonstrated that the back gate allows reducing the subthreshold leakage current, improving the subthreshold slope and adjusting the threshold voltage. At certain back gate voltage, transistors operate as typical normally-off devices. Multi-gate grating gate transistors of high gate area—0.25 mm${}^{2}$—and total channel area—0.64 mm${}^{2}$—were fabricated for the purpose of the experiments at terahertz frequencies. In spite of the extremely high gate area, transistors demonstrated small subthreshold leakage current, which is a requirement for high frequency experiments. The subthreshold leakage current can be further reduced by the back gate voltage. This study confirmed the feasibility of the approach for fabricating high-area AlGaN/GaN HEMTs with small gate leakage current. Although the computer simulation predicted different electron density distributions while operating the transistor by either top or back gates, the low frequency noise measurements indicated identical noise properties in these two modes of operation. This is a proof that the dominant mechanism of the low frequency noise in AlGaN/GaN HEMTs is tunneling of electrons to the traps in the AlGaN barrier layer. The trap density extracted from the noise measurements was found to be the same as or lower than that reported earlier for other GaN-based transistors. Therefore, we conclude that complications related to the fabrication of double-quantum-well structures did not deteriorate the noise properties.

## Abbreviations

The following abbreviations are used in this manuscript:

HEMT	High Electron Mobility Transistor
FinHEMT	Fin-Shaped High Electron Mobility Transistor
MOSFET	Metal-Oxide-Semiconductor Field-Effect Transistor
